# Genotype–Phenotype Relationships in Patients Carrying Biallelic *FDXR* Pathogenic Variants—New Cases and Systematic Review

**DOI:** 10.1155/humu/5928629

**Published:** 2026-10-02

**Authors:** Antoine Paul, Ghizlene Lahlou, Sophie Achard, Jérôme Nevoux, Isabelle Mosnier, Isabelle Rouillon, Yann Nguyen, Maya Benali, Jean-François Papon, Agnes Rotig, Margaux Serey-Gaut, Celine Labeyrie, Guillemette Beaudonnet, Laurence Jonard, Ralyath Balogoun, Sandrine Marlin

**Affiliations:** ^1^ Department of Otolaryngology Bicetre Hospital APHP Le Kremlin-Bicêtre France; ^2^ Department of Otolaryngology and Auditory Implants Pitie-Salpetriere Hospital AP-HP Sorbonne University Paris France sorbonne-universites.fr; ^3^ Audiology Research Center Pitie-Salpetriere Hospital APHP Paris Cite University Pasteur Institute Inserm CNRS Fondation pour l′audition IHU reConnect Paris France cnrs.fr; ^4^ CRMR surdites genetiques Pitie-Salpetriere Hospital APHP Paris France; ^5^ Department of Pediatric Otolaryngology Necker-Enfants Malades Hospital APHP Paris France; ^6^ Department of Ophthalmology Bicetre Hospital APHP CRMR OPHTARA Paris-Saclay University Paris France universite-paris-saclay.fr; ^7^ Genetics of Rare Ophthalmological Auditory and Mitochondrial Disorders Inserm UMR S1163 Imagine Institute Paris France; ^8^ Reference Center for Genetic Hearing Loss Department of Genomic Medicine for Rare Diseases Necker-Enfants Malades Hospital APHP Paris France; ^9^ Department of Neurology French National Reference Center for Rare Neuropathies Bicetre Hospital APHP Le Kremlin-Bicêtre France

**Keywords:** deafness, *FDXR*, ferredoxin reductase, genetics, neuropathy, review

## Abstract

Biallelic pathogenic *FDXR* variations were identified in 2017 as being responsible for sensorial neuropathies. The first reported patients suffered from auditory and optic sensorineural impairments (ANOA, MIM #617717). Since then, many publications have described various phenotypes including peripheral and central nervous systems impairments (MMDS9B, MIM #620887). This is consistent with the role of the encoded protein ferredoxin reductase (FDXR) as one of the actors of iron‐sulfur proteins synthesis in mitochondria. Ophthalmologic and neurologic symptoms have often been reported, but little is known about the associated inner ear disabilities, although hearing loss was described in the early clinical reports. Firstly, we would like to characterize inner ear abnormalities, sharing our observations of five new patients′ hearing and the outcome after rehabilitation. Thereafter, considering the increasing numbers of publications about *FDXR*, it appeared useful to provide an overview of literature, analyzing how molecular data may correlate with clinical symptoms. We analyzed the clinical and molecular findings in patients reported from 2017 until March 2025. Data included 89 cases from 79 unrelated families. Protein changes were mostly located in the FAD binding domain, whereas those in NADPH binding domains seem to be less prevalent but more related to severe cases with earlier onset symptoms. These data, combined with current knowledge about FDXR catalytic function and its interaction with FDX and/or cytochrome P450 proteins, could help to understand potential phenotype‐genotype correlations.

## 1. Introduction

Rare genetic etiologies of sensorial phenotypes and associated optic and auditory disorders have been identified to date. Pathogenic variants of *OPA1* (OMIM: 605290) or *TMEM126A* genes (OMIM: 612988) lead to isolated sensorial impairments, whereas neurological symptoms could be associated with other genetic diseases such as in the Brown Vialetto Van Laere syndrome (OMIM: 211530), Friedreich ataxia (OMIM: 229300), or Mohr‐Tranebjaerg syndrome (OMIM: 304700). In 2017, we first demonstrated the involvement of *FDXR* in an association of sensorial neuropathies (OMIM: 617717) [1]. Hearing loss (HL) and ophthalmological (OPH) signs, mostly corresponding to optic atrophy (OA), and/or retinal dystrophy (RD), have been observed. However, publications in the last few years have shown evidence that sensorial impairments are only part of broader phenotypes including, to varying degrees, not only central and peripheral nervous system (CNS, PNS) impairments, but also genital abnormalities.

Despite improving knowledge about FDXR phenotypes, little has been said about HL and the rehabilitation outcomes. HL observed in these patients likely corresponds to auditory neuropathy spectrum disorders (ANSD) defined by normal outer hair cell function (valid otoacoustic emission responses and/or detection of cochlear microphonic potentials) associated with the absence of identifiable synchronized auditory brainstem responses (ABR). It is therefore interesting to focus on inner ear disabilities by sharing our recent experience. In this context, we described five new cases from four unrelated families, highlighting their hearing history and rehabilitation results. Each patient received written and oral information about this study. This report complies with the Helsinki declaration and was approved by the ethical committee of the French Society of Otolaryngology (N°2025‐01‐052‐AP).

Secondly, we attempted to integrate the recent increasing data about *FDXR* phenotypes with our knowledge about the encoded flavoprotein called ferredoxin reductase (FDXR). Before first clinical findings were reported, FDXR was already known to play a role in mitochondria redox reactions, allowing the synthesis of iron‐sulfur proteins (ISP) particularly involved in mitochondrial respiratory complexes (MRC) assembly as well as iron homeostasis [2]. FDXR catalyzes the ferredoxin protein (FDX) reduction by transferring electrons originating from nicotinamide adenine dinucleotide phosphate (NADPH) enzyme. FDXR–FDX molecular interactions have been heavily studied in the past two decades [3, 4] as FDX′s role is critical for additional redox reactions with iron‐sulfur scaffolds from which ISP are produced. In addition, the proper functioning of FDXR‐FDX redox in adrenal cells can enable hormonal synthesis as well, involving cytochrome P450 enzymes [5, 6]. On the other hand, Frataxin protein, encoded by *FXN* implicated in Friedreich ataxia (FA), is also one of the ISP synthesis actors providing iron fixation early in the assembly process [2]. This common metabolic pathway may explain overlapping features between FA and FDXR phenotypes.

Thus, it seemed relevant to analyze how protein changes (Pc) are predicted to influence FDXR function or synthesis. Some of them may interfere more or less with its binding and catalytic sites related to FDX protein or NAPDH enzyme. We have therefore dedicated the second part of this work to provide further insight into the correlation between molecular and clinical data, based on an updated and exhaustive literature review.

## 2. Clinical Reports

### 2.1. Patient 1

A female Caucasian patient, with no family history, presented with progressive visual acuity loss since the age of 6. Rod‐cone RD was diagnosed at the age of 16. Her visual acuity was 9/10 P2 on both eyes with a preserved central visual field and normal color vision testing. An important decrease of vision was observed at the age of 20, resulting in a visual acuity at hand motion on both eyes. Binocular kinetic visual field testing showed a small residual island on the right side with central vision loss. Fundus examination revealed severe optic disc atrophy, vessel narrowing and bone‐spicule‐like retinal pigmentary changes. Macular optic coherence tomography (OCT) showed severe inner and outer retinal layer thinning; optic nerve (ON) pallor led to the diagnosis of bilateral OA. She had no other medical history. There was no family history. Metabolic assessments were negative. At the age of 23, she was referred to the otolaryngology department for acute mild HL. Three months after, speech intelligibility was severely impaired. She presented with bilateral mild to moderate sensorineural HL on pure tone average (PTA), whereas speech recognition score did not exceed 40% at 40 dB on the left ear with severe distortion; no intelligibility remained on the right ear. PTA worsened progressively on both sides reaching a moderate/severe level of HL (Figure S1). Brain and auditory pathways MRI were normal. Transient otoacoustic emissions (TEOAE) were obtained on both ears, whereas wave V was hardly identifiable at 90 dB HL when testing ABR with dyssynchronous shape of neuronal responses. Caloric tests revealed bilateral hyporeflexia. She also complained of bilateral upper limb tingling associated with pyramidal syndrome and bilateral Babinski sign. Slight ataxia with hypopallesthesia at the ankles and positive Romberg sign were observed. Nerve conduction studies disclosed bilateral low compound muscle action potential amplitudes on common fibular motor nerve. Somatosensory evoked potential disclosed medullary neurophysiological involvement.

Trio genome sequencing revealed two compound heterozygous *FDXR* missense variants: NM_24417.5 c.1427G>C, p.R476T (highly damaging) and c.1361 C>T p.S454F (probably damaging), both expected to be highly damaging, inherited from her mother and father, respectively.

She underwent right cochlear implantation 6 months after HL was diagnosed (Medel brand, model: Synchrony2‐Flex28). Good discrimination improvement was observed 1 month after surgery when fitting CI and contralateral hearing aid (HA): Speech recognition threshold (SRT) = 60 dB SPL. However, understanding skills progressively decreased in the following year: At the 12th postoperative month, PTA was stable, whereas speech needed to be slow and at a short distance. Contralateral implantation was performed 18 months after the first implantation. Speech discrimination recovered during the first few months, but gradually declined until reaching a stable level at the 6th postoperative month. Because of facial nerve stimulation, pulses were switched from biphasic to triphasic signals associated with HDCIS strategy with a reduced frequency stimulation. Despite these programming adjustments, the patient suffered from loudness, limiting CI use to quiet environments. The patient reported benefits from CIs allowing better voice recognition and sound localization, whereas conversation remained difficult, still requiring braille communication. Thus, datalogging showed only few hours of CI use per day.

### 2.2. Patients 2 and 3 (Siblings)

In this consanguineous Algerian family, Patient 2 complained about HL at the age of 28. He suffered from tinnitus and understanding trouble with poor outcome when wearing HAs. He presented with bilateral mild to moderate sensorineural HL. Discrepancies between PTA and speech recognition were obvious in both ears. OAE showed clear valid responses on both sides, whereas ABR could not identify synchronous responses (Figure S2). Caloric tests showed bilateral vestibular hypofunction with low angular velocity. He did not complain of dizziness or gait difficulty. Cerebral and auditory pathways were normal on MRI. No vision loss was reported; OPH assessment revealed asymptomatic OA with temporal retinal pallor and thickening on OCT and eye fundus. Electroretinogram (ERG) and visual‐evoked potential (VEP) were normal.

A homozygous *FDXR* pathogenic variant was identified by targeted sequencing of ANSD genes: NM_24417.5 c.916C>T p.R306C (damaging), recurrently observed in this case series (Patients 2, 3 and 4) and in previous publications [1, 7, 8].

The patient increasingly relied on lipreading as hearing progressively worsened over 1 year. Cochlear implantation was performed (Advances Bionics brand, Hires Ultra3D‐SlimJ). Autosense system was used. In spite of intelligibility improvement (*S* *R* *T* = 25 *d* *B*), sound distortion above 50 dB was noted. However, CI was daily used (the last datalogging showed more than 8 h of CI use); slow conversation was necessary for the patient to be able to understand.

His elder brother (Patient 3) presented with HL at the age of 31, carrying the same homozygous variant. At his first visit, he complained of understanding difficulties in the left ear with increasing tinnitus and vertigo episodes. He presented with mild and moderate sensorineural HL in right and left ears, respectively. No intelligibility remained on the left side contrary to the right ear. Three years later, bilateral moderate SNHL was observed, with uncorrelated bilateral poor speech recognition scores. OAE was also obtained, whereas no neuronal response was identifiable on ABR (Figure S3). Caloric tests, vHIT and inner ear imaging were normal. The patient did not complain of visual loss but no ophthalmologic assessment has been performed so far. He declined cochlear implantation.

### 2.3. Patient 4

A female patient of Moroccan origin suffered from bilateral HL with tinnitus at the age of 18. No vertigo was reported. HAs were irregularly used due to their mild understanding benefit. Hearing worsened progressively over the following 2 years, until the complete absence of intelligibility. OAE was obtained whereas shape of unsynchronized neuronal responses was observed on ABR (Figure S4). She also complained of hyposensitivity in the lower limbs with hypopallesthesia and tingling, though somesthesis‐evoked potentials were normal. Cerebral and auditory pathways were normal on MRI. Asymptomatic optic neuropathy was diagnosed with bilateral temporal pallor on eye fundus. Crystal inclusion in the anterior eye chamber was observed. OCT was normal and ERG revealed bilateral decreased retinal electrogenesis in scotopic and photopic conditions, as well as bilateral axonal deficit.

Compound heterozygous *FDXR* pathogenic variants were identified by sequencing specific ANSD genes panel: NM_24417.5 c.487G>A p.G163R (probably damaging), which has never been reported to date but considered as possibly damaging, and c.916C>T (p.R306C) as observed in Patients 2 and 3.

Cochlear implantation was suggested but the patient declined. Follow‐up is ongoing.

### 2.4. Patient 5

A female patient, of Moroccan origin, had been suffering from HL since the age of 14. Her parents are first cousins and HL was reported in two maternal great‐uncles, one of them having two daughters suffering from HL as well. HA fitting was attempted at 16 because of increasing scholarship difficulties, with few benefits. Frequent falls and progressive visual acuity loss have appeared in the meanwhile. Audiometry disclosed sensorineural moderate HL on PTA with rising curve, whereas intelligibility was inconsistently worse. ABR were performed revealing unidentifiable responses on both ears. Valid OAE could be recorded on the right ear but not on the left; cochlear microphonic potentials (CMPs) measurement was therefore performed. CMPs could be recorded at 70 and 90 dB on the left and right ears, respectively (Figure S5). Vestibular caloric and vHIT tests were normal. Cerebral and auditory pathways MRI was normal. Visual acuity was assessed: 4/10 and 6/10, respectively, on the right and left eyes. Bilateral temporal OA was diagnosed in both eyes associated with central scotoma and dyschromatopsia (Verriest Axe III). Neurologic assessment revealed feet deficit for dorsal flexion in the feet, bilateral positive Babinski signs, abolition of patellar tendon and Achilles osteotendinous reflexes. Electroneuromyography (ENMG) revealed decreased amplitude of motor and sensitive responses with normal latencies in the lower limbs corresponding to axonal anomalies. Sudoscan (measuring electrochemical skin conductance) highlighted abnormalities of the sensitive little fibers. Targeted ANSD genes identified homozygous *FDXR* variant: NM_24417.5 c.1120G>A p.D374N (damaging) already reported [8].

As hearing worsened, bilateral sequential cochlear implantation (Cochlear brand, Nucleus CI522) was performed at the age of 18 with initial satisfactory outcome. At the sixth postoperative month, average PTA with both CI was 20 dB, whereas SRT was 35 dB with a maximum score of 80% at 60 dB. Intelligibility decreased over the following 2 years, word recognition maximum score hardly reached 40% at 70 dB. The last evaluation disclosed 78% and 67% of word and sentence recognition, respectively, in silence, with worse scores in noise (SNR 10 dB: 38% and 13%). At the 4th postoperative year, she displayed poor visual acuity: 2/10 and 3/10 in the right and left eyes, respectively. Consequent to both gait disability and visual loss, she could hardly move without a cane. In addition, behavior troubles progressively appeared with hallucinations. On the last programming session, datalogging showed 11.6 h of CI use; ACE strategy was used. Because of sound distortion and loudness, pulses were enlarged with decreased frequency of stimulation (500 Hz), maxima was set to 5.

All patients were born before 2000, as neonatal hearing screening was introduced in France only in 2012; no data about neonatal screening was available. Main clinical and molecular data are summarized in Table 1.

**Table 1:** Summary of clinical findings in patients of the current report.

	Patient 1	Patient 2	Patient 3	Patient 4	Patient 5
Nucleotide variants	c.1427G>C ClinPred = 0.996 (highly damaging) Sift = 0.0 (damaging)	c.916C>T (Ho) ClinPred = 0.612 Sift = 0.028 (damaging)		c.487G>A ClinPred = 0.999 (probably damaging) Sift = 0.0 (damaging)	c.1120G>A (Ho) ClinPred = 0.988 (damaging) Sift = 0.007 (damaging)
	c.1361 C>T ClinPred = 0.988 (probably damaging) Sift = 0.002 (damaging)			c.916C > T ClinPred = 0.612 Sift = 0.028 (damaging)	
Protein changes	p.R476T/p.S454F	p.R306C	p.R306C	p.G163R/p.R306C	p.D374N
Hearing loss onset	23 years	28 years	31 years	18 years	14 years
ANSD	Yes	Yes	Yes	Yes	Yes
Auditory rehabilitation	CI	CI	HA	HA	CI
Vision loss onset	6 years	No visual loss	No visual loss	No visual loss	14 years
Ophthalmological signs	Visual acuity loss, RD, reduced VF, optic disc atrophy	Asymptomatic OA Temporal RD	NA	Asymptomatic	Visual acuity loss, temporal OA, central scotoma, dyschromatopsia
Neurological signs	Hypopallesthesia, tingling	None	None	Hypopallesthesia, tingling	Abolition of OTR, pyramidal signs, lower limbs motor deficit

*Note:* All nucleotide variants were indicated according to NM_24417.5 nomenclature.

Abbreviations: ANSD, auditory neuropathy spectrum disorder; CI, cochlear implantation; HA, hearing aids; Ho, homozygous variant; OA, optic atrophy; OTR, osteotendinous reflexes; RD, retinal dystrophy; VF, visual field.

## 3. Literature Review

### 3.1. Methods and Statistical Analysis

We reviewed all cases published from 2017 until March 2025 (as this manuscript has been submitted in April 2025) with biallelic *FDXR* pathogenic variations [1, 5, 7–21].

Each predicted Pc location was noted and classified with respect to the affected binding domain (Figure 1). In this study, NADPH binding domains were named NADPH1 and NADPH2, respectively, for the N and C‐terminal domains, whereas FAD/NADPH binding domain was named FAD for the sake of clarity. The mitochondrial targeted signal was named MTS.

**Figure 1:**
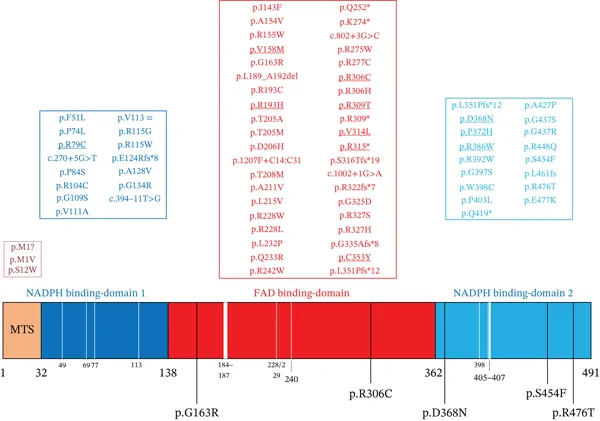
Location of the predicted protein changes reported up to March 2025. Predicted protein changes carried by the patients described in this report are highlighted below the protein schema. Domain annotation is based on a crystallography analysis of the *Bos taurus* FDXR ortholog as illustrated by previous authors [5, 10]. White bars correspond to catalytic sites (Uniprot database). Recurrent protein changes were underlined. MTS: mitochondrial targeted signal.

All variants were indicated according to the NM_024417.5 nomenclature; some of them may appear differently from the original publication [8, 12]. All phenotypes were studied, and the main symptoms were classified as CNS or PNS impairments, HL or OPH signs. As nystagmus was difficult to assign to vestibular, CNS or OPH impairments, it has been reported separately. Other clinical findings were detailed separately as well. We have distinguished between two groups of phenotype severity: “severe phenotype” and “milder phenotype.” Those considered as severe corresponded to patients presenting with one of the following symptoms: gait ataxia, hypotonia, developmental delay, delayed motor development, premature death, microcephaly, encephalopathy, spastic gait, regression of motor milestones, seizure, cognitive loss, intellectual delay, psychiatric disorder, developmental regression, paralysis with loss of trunk control, intellectual disability, cerebellar atrophy, encephalitis, neurological disease in childhood, and genital anomalies.

We classified patients carrying biallelic missense variants regarding the predicted Pc position:

- NADPH1, FAD, and NADPH2 groups for patients carrying Pc exclusively located in one of these domains.
- NADPH1 + FAD, FAD+NADPH2, and NADPH1 + 2 groups include those with Pc located in the two corresponding binding‐domains.
- MTS group for patients carrying at least one Pc in MTS domain.

Age onset was classified in three groups: from birth to 5 years old, from 6 to 15 years old, and above 15 years old (named “birth–5y”, “6–15y” and “> 15y,” respectively). No average age could be calculated, as the exact age onset has not been systematically specified. This classification allowed differentiation between onset in infancy/toddler, childhood/adolescence, or adulthood (from the end period of pubertal growth), respectively.

Distribution of affected binding domain groups was analyzed according to the phenotype severity, the age onset, and the clinical findings. Pc positions were studied with respect to FDXR catalytic sites reported in Uniprot databases. We also identified variations observed recurrently, corresponding to those reported in nonconsanguineous probands, either described within one same publication with no family relationship specified or reported by different teams.

Analysis was done comparing independent variables (binding‐domains, age onset, main clinical findings, phenotype severity according to the groups described previously). Statistical analysis was done with Fisher′s exact test and Pearson′s chi‐squared test depending on the group size (below or above five cases). A *p* value below 0.05 was considered statistically significant.

### 3.2. Results

Until March 2025, 89 cases from 79 unrelated families have been described. Table 2 summarizes the main clinical signs, predicted Pc and corresponding affected binding‐domains observed in each patient.

**Table 2:** Overview of clinical and molecular data in patients reported up to March 2025.

Authors	Cases	PC1	PC2	MTS	NADPH1	FAD	NADPH2	Age at HL confirmation	CNS	PNS	OPH	HL	Ntg
Paul et al. 2017	1^a^	p.R306C	p.R306C	—	—	+	—	13 years	—	—	OA	+	—
	2^a^	p.R306C	p.R306C	—	—	+	—	13 years	—	—	OA	+	—
	3^a^	p.R306C	p.R306C	—	—	+	—	15 years	—	—	OA	+	—
	4^a^	p.R306C	p.R306C	—	—	+	—	11 years	—	—	OA	+	—
	5^a^	p.R306C	p.Q419^a^	—	—	+	+	Teenage	—	—	OA	+	—
	6^a^	p.R242W	p.R327S	—	—	+	—	2 years	—	+	OA/RD	+	—
	7^a^	p.L215V	p.E477K	—	—	+	+	Childhood	—	—	OA	+	—
	8^a^	p.L215V	p.E477K	—	—	+	+	5 years	—	—	OA	+	—
Peng et al. 2017	1^b^	p.R386W	p.R386W	—	—	—	+	Childhood	+	—	OA	—	—
	2^b^	p.R386W	p.R386W	—	—	—	+	Childhood	+	—	OA/RD	—	—
	3^b^	p.F51L	p.G437S	—	+	—	+	2 years	+	+	RD	—	+
	4^b^	p.R386W	p.R386W	—	—	—	+	Congenital	+	+	—	—	—
	5^b^	p.R386W	p.R386W	—	—	—	+	Childhood	+	—	OA	+	—
	6^b^	p.P74L	p.R386W	—	+	—	+	Childhood	+	+	OA	—	—
	7^b^	p.I143F	p.C353Y	—	—	+	—	1 year	+	—	OA	—	—
	8^b^	p.T205A	p.G437S	—	—	+	+	3 years	+	—	OA	—	—
	9^b^	p.D368N	p.D368N	—	—	—	+	8 years	+	+	+	+	+
	10^b^	p.D368N	p.D368N	—	—	—	+	4 years	+	+	+	+	+
	11^b^	p.V158M	p.I207F	—	—	+	—	Childhood	+	+	OA/RD	+	—
	12^b^	p.R315 ^∗^	p.P403L	—	—	+	—	Childhood	+	+	+	—	—
	13^b^	p.R386W	p.R386W	—	—	—	+	Childhood	+	—	OA	—	—
	14^b^	p.V158M	p.K274 ^∗^	—	—	+	—	2.5 years	+	+	OA	+	+
	15^b^	p.R386W	p.R386W	—	—	—	+	Childhood	+	—	OA/RD	—	—
Slone et al. 2018	1^b^	p.M1?	p.R155W	+	—	+	—	Childhood	+	—	OA/RD	—	—
	2^b^	p.R193H	p.S316Tfs ^∗^19	—	—	+	—	Childhood	+	—	OA	—	—
Stenton et al. 2020	1^b^	p.S12W	p.R228L	+	—	+	—	Childhood	+	+	OA	+	—
	2^b^	p.V111A	p.L189_A192del	—	+	+	—	7 months	+	+	OA	—	+
	3^b^	p.G109S	p.R448Q	—	+	—	+	4 years	+	—	—	+	—
	4^b^	p.G109S	p.R448Q	—	+	—	+	4 years	+	—	—	—	—
	5^b^	p.R228L	p.C353Y	—	—	+	—	2 years	+	—	OA	+	—
	6^b^	p.A211V	p.A211V	—	—	+	—	4 years	+	+	OA	+	—
	7^b^	p.R386W	p.R386W	—	—	—	+	5 months	+	+	OA/RD	—	—
Yang et al. 2021	1^b^	p.P84S	p.D206H	—	+	+	—	9 months	+	—	OA/RD	+	—
Keller et al. 2021	1^b^	p.R155W	p.R155W	—	—	+	—	4 years	+	+	OA	—	—
Song et al. 2021	1^a^	p.V314L	p.D368N	—	—	+	+	12 years	—	—	OA	—	—
Jukurte et al. 2021	1^a^	p.R193C	p.P372H	—	—	+	+	3 years	—	+	OA/RD	—	+
	2^a^	p.R315^a^	p.P372H	—	—	+	—	14 years	—	+	OA/RD	+	—
	3^a^	p.R315^a^	p.P372H	—	—	+	—	4 years	—	+	OA/RD	+	—
	4^a^	p.A154V	p.R275W	—	—	+	—	7 years	—	—	OA/RD	+	+
	5^b^	p.P372H	p.A427P	—	—	—	+	22 years	—	+	OA	—	—
	6^b^	p.T205M	p.R275W	—	—	+	—	Childhood	+	+	OA	+	—
	7^b^	p.P372H	chr17del^a^	—	+	+	+	Childhood	+	—	OA/RD	—	+
	8^b^	p.P372H	p.G397S	—	—	—	+	3.5 years	+	+	OA/RD	—	—
	9^b^	p.P372H	p.G397S	—	—	—	+	2.5 years	—	+	OA	—	—
	10^a^	p.R242W	p.R306C	—	—	+	—	42 years	—	+	OA/RD	—	—
Masnada et al. 2023	1^a^	p.R155W	p.R155W	—	—	+	—	9 years	—	+	ON/R	+	—
	2^b^	p.R155W	p.R155W	—	—	+	—	7 years	+	+	OA	—	—
Yi et al. 2023	1^b^	p.R193H	p.R193H	—	—	+	—	Birth	+	—	OA/RD	—	+
	2^b^	p.R79C	p.R104C	—	+	—	—	Birth	+	—	OA/RD	—	+
	3^b^	p.G134R	p.R306H	—	+	+	—	Birth	+	—	OA/RD	—	+
	4^a^	p.R115G	p.Q233R	—	+	+	—	Childhood	—	—	OA/RD	—	+
	5^a^	p.R228W	p.V314L	—	—	+	—	Childhood	—	—	OA/RD	—	—
	6^a^	p.R104C	p.R104C	—	+	—	—	Childhood	—	—	OA/RD	—	—
	7^a^	p.R115G	p.G325D	—	+	+	—	Childhood	—	—	OA/RD	—	—
	8^a^	p.L232P	p.R275W	—	—	+	—	5 years	—	—	OA/RD	—	+
	9^a^	p.R104C	p.W398C	—	+	—	+	3 years	—	—	OA/RD	—	—
	10^a^	p.R79C	p.V314L	—	+	+	—	6 years	—	—	OA/RD	—	—
	11^a^	p.R79C	p.V314L	—	+	+	—	3 years	—	—	OA/RD	—	—
Wei et al. 2023	1^b^	p.A128V	p.R322fs ^∗^7	—	—	+	—	1 years	+	—	OA/RD	+	—
	2^b^	p.V314L	c.394‐11 T > G	—	+	+	—	10 years	+	—	OA/RD	—	—
	3^a^	p.V314L	p.L351Pfs ^∗^12	—	—	+	—	5 years	—	—	OA/RD	—	—
	4^b^	c.1002+1G>A	p.R306C	—	—	+	—	3 years	—	—	OA/RD	+	—
	5^b^	p.V158M	p.R193C	—	—	+	—	4 years	—	—	OA/RD	—	—
Kim et al. 2024	1^a^	p.V314L	p.V314L	—	—	+	—	Childhood	—	—	OA/RD	+	—
Campbell et al. 2024	1^b^	p.R386W	p.R386W	—	—	—	+	4 months	+	—	OA	—	—
	2^b^	p.R386W	p.P403L	—	—	—	+	5 months	+	—	+	+	+
	3^b^	p.R309T	p.P403L	—	—	+	+	9 months	+	—	OA/RD	—	+
	4^b^	p.R309T	p.P403L	—	—	+	+	9 months	+	—	+	—	+
	5^b^	p.G335Afs ^∗^8	p.G397S	—	—	+	—	18 months	+	—	+	—	+
	6^a^	p.R306C	p.R306C	—	—	+	—	7 years	—	—	+	+	—
	7^b^	p.M1V	p.D368N	+	—	—	+	2.5 years	+	—	+	+	+
	8^b^	p.R386W	p.R386W	—	—	—	+	3 months	+	—	+	—	+
	9^b^	c.270+5G>T	p.R386W	—	+	—	+	4 months	+	—	+	—	—
	10^b^	p.R386W	p.R386W	—	—	—	+	4 months	+	—	+	+	+
	11^b^	p.R115W	c.802+3G>C	—	—	+	—	9 months	+	—	R	—	—
	12^a^	p.R327H	p.L461fs	—	—	+	+	21 years	—	—	R	—	—
	13^b^	p.R386W	p.G397S	—	—	—	+	3 months	+	—	+	+	—
	14^b^	p.V113=	p.Q252^a^	—	—	+	—	6 months	+	—	+	—	+
Pignatti et al. 2024	1^b^	p.G437R	p.G437R	—	—	—	+	Infant	+	+	OA	—	—
	2^b^	p.G437R	p.G437R	—	—	—	+	Infant	+	+	—	—	—
Kaler and Couser 2024	1^b^	p.R193H	p.R277C	—	—	+	—	Adolescence	+	+	—	+	+
Cao et al. 2025	1^b^	p.E124Rfs ^∗^8	p.T208M	—	—	+	—	16 years	+	—	+	—	+
	2^a^	p.W160L	p.V314L	—	—	+	—	10 years	—	—	+	—	—
Wang et al. 2025	1^b^	p.R309^a^	p.V314L										
Current reports	1^a^	p.S454F	p.R476T	—	—	—	+	6 years	—	+	OA/RD	+	—
	2^a^	p.R306C	p.R306C	—	—	+	—	28 years	—	—	OA/RD	+	—
	3^a^	p.R306C	p.R306C	—	—	+	—	31 years	—	—	—	+	—
	4^a^	p.G163R	p.R306C	—	—	+	—	18 years	—	+	OA	+	—
	5^b^	p.D368N	p.D368N	—	—	—	+	14 years	+	+	OA	+	—

*Note:* Age at HL confirmation: infant, childhood or adolescence were indicated when the exact age was not available. Binding domains affected by intronic variants were determined according to the presumed splice sites and/or exon endpoint localizations. Among patients carrying nonsense or truncating frameshift variants, the affected binding domains were determined according to the associated missense variant position.

Abbreviations: CNS/PNS, central/peripheral nervous system; HL, hearing loss; NTG, nystagmus; OA, optic atrophy; OPH, ophthalmological symptoms; PC, protein change; RD, retinal dystrophy.

^a^Milder phenotype.

^b^Severe phenotype.

Seventy‐six distinct Pc have been identified so far; Figure 1 indicates their location according to FDXR binding domains. Pc reported up to date resulted from 58 missense, 6 frameshift, 5 nonsense, and 4 intronic variants. In addition, we have noted two deletions, one corresponded to a large chromosomic deletion (chr17: 74818633—74888183del), and one with four codon deletion p.L189_A192del. One synonymous variant has been reported: c.339G>A (p.V113=) [18]. Patients carrying biallelic missense variants account for the majority part of this cohort (*n* = 70). There were no biallelic nonsense or truncating frameshift variants, each of which was associated with one missense variant.

The most frequently reported clinical findings were: OPH signs, CNS or PNS impairments, and HL; observed in 83, 53, 31, and 38 cases, respectively. Fifty‐seven patients were classified as severe whereas 32 were considered as presenting with milder phenotype. Detailed phenotypes have been displayed in the supplemental material (Table S1) reporting clinical findings as close as possible to the authors descriptions (with partly reproduced wording) and specifying how symptoms were categorized. In most cases, age of symptom onset was reported within the first years of life, in the period “birth to 5 years‐old” (57 patients), followed by those with symptoms appearing in the “6–15 years old” period or during adulthood (25 and 7 patients, respectively) (Table 3).

**Table 3:** Summary of the main clinical and genetic findings.

	*n* (%)
**All cases**	**89**
**Clinical findings**	
Ophthalmological	83 (93)
Central nervous system	53 (60)
Hearing loss	38 (43)
Peripheral nervous system	31 (35)
Nystagmus	22 (25)
Genital anomalies/steroid hormonal defect	4 (4)
**Phenotype**	
Severe phenotype	57 (63)
Milder phenotype	32 (37)
**Age onset**	
Birth–5 years	57 (64)
6–15 years	25 (28)
> 15 years	7 (8)
**Pathogenic variants**	
Biallelic missense	70 (79)
Nonsense + missense	7 (8)
Frameshift + missense	6 (7)
Intronic + missense	4 (5)
Deletion + missense	2 (1)

Abbreviations: M, milder phenotype; S, severe phenotype.

A preliminary phenotype severity comparison was done between patients carrying biallelic missense variants from NADPH1, FAD and NADPH2 groups on one hand, and those with nonsense or truncating frameshift variants on the other hand. In these last, classification was made only considering the associated missense variant position. Similarities in mild/severe phenotype ratio were noticed: The FAD domain group was more related to milder phenotype contrary to the NADPH2 group (Table S2). Thus, patients with nonsense or truncating frameshift variants were included in all the following binding‐domain groups analysis, classifying each of them in the NADPH1, FAD, or NADPH2 groups according to the missense variant with which they are associated.

FAD binding‐domain was impacted most often, as FAD group included 32 patients, followed by patients in NADPH2, FAD + NADPH2, and NADPH1 + FAD groups (25, 7, and 6 patients, respectively). Patients in NADPH1 + 2, NADPH1, and MTS groups were the least observed: five, four, and three cases, respectively (Figure 2). Pc on one catalytic site, position 228, was observed in only three patients: unrelated Patients 1 and 5 in Stenton et al.′s study [11], and Patient 5 reported by Yi et al. [16]. However, resulting amino acids were different (p.R228L and p.R228W).

**Figure 2:**
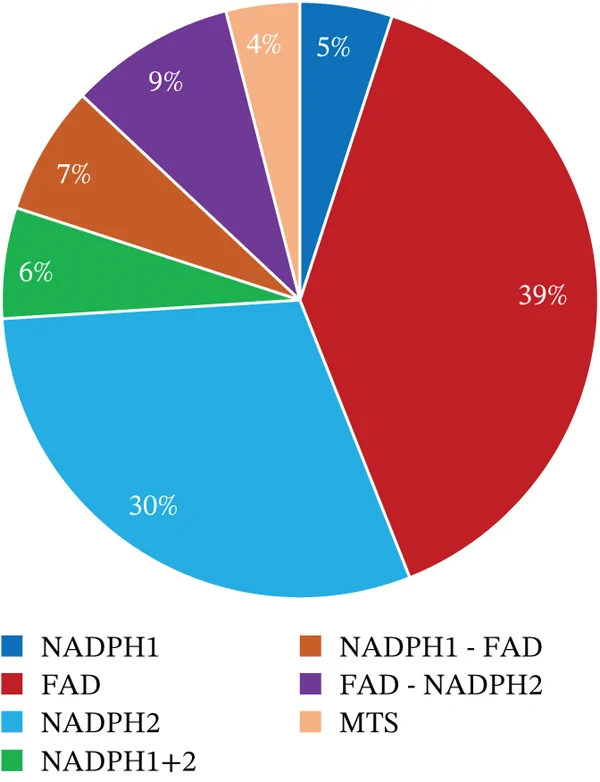
Distribution of patients according to the binding‐domain groups. Data were analyzed among patients carrying either biallelic missense variants or truncating associated with missense variants.

When analyzing patients according to the phenotype severity (Figure 3A), we observed the following:

- Patients in the FAD group were significantly more likely to present with a milder phenotype: 18/31 (58%) versus 14/51 (27%) in the mild and severe groups, respectively, *p* = 0.006.
- On the other hand, patients in the NADPH2 were more likely to have a severe phenotype: 22/51 (43%) versus 3/31 (10%) in the severe and mild groups, respectively, *p* = 0.001.
- For the other subgroups, we observed a more severe phenotype in the NADPH1, NADPH1 + 2, and MTS group and a milder phenotype in the NADPH1 + FAD and FAD + NADPH2 phenotype but these differences were not significant.

**Figure 3:**
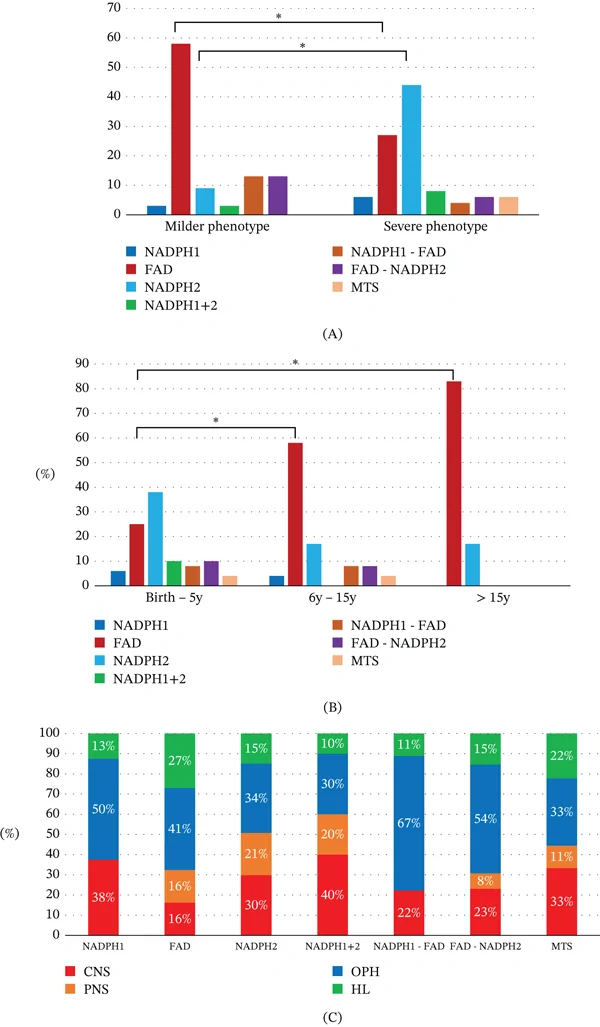
Distribution of the main clinical findings according to the affected binding‐domain group. (A) Distribution of binding‐domain groups according to the phenotype severity, (B) distribution of binding‐domain groups according to age onset, and (C) distribution of clinical findings according to the binding domains group. CNS/PNS, central or peripheral nervous system; HL, hearing loss; OPH, ophthalmological signs.

Looking at the age of onset (Figure 3B):

- The first symptoms tend to appear at an older age in the FAD group patients.
- The proportion of FAD group patients was significantly lower in the birth–5 years group (13/52, 25%) compared with the 6–15 years (14/24, 58%, *p* = 0.009) and > 15 years (5/6, 83%, *p* = 0.01) groups.
- In the > 15 years group, PC was observed neither in NADPH1 nor in MTS domains
- NADPH1 + 2 patients were observed in the “birth‐5y” group only.

When analyzing clinical findings (Figure 3C):

- OPH signs were the most prevalent findings in all binding‐domain groups except for NADPH1 + 2.
- Proportion of CNS impairment was higher in NADPH2, NADPH1, MTS, and NADPH1 + 2 groups (40%, 38%, 33%, and 30%, respectively), as compared with those in FAD + NADPH2, NADPH1 + FAD, and FAD groups (16%, 22%, and 23%)
- HL was more prevalent than CNS impairment only in the FAD group.

Twelve recurrently predicted Pc were noted, 7 in FAD, 4 in NADPH2, and 1 in NADPH1 binding domains, each was induced by the same nucleotide variant. Table 4 displays all of them indicating cases in whom they were identified. The most recurrently observed were as follows:

- p.R386W (NADPH2): 14 cases from 13 families [8, 11, 18]. Nine of them carried homozygous variants. All of them presented with severe phenotype.
- p.R306C (FAD): nine cases from five families including our current report [1, 9, 18]. Two cases carried compound heterozygous variants, whereas the remaining carried homozygous variants. All but one (Campbell′s Patient 4) were categorized in the “milder phenotype” group.
- p.V314L (FAD): nine unrelated patients [9, 14, 16, 17, 20, 21], all carried compound heterozygous variants except for Kim et al.′s patient. All but two (patients from Wei and Wang′s studies) corresponded to “milder phenotypes.”
- p.P372H (NADPH2): Seven cases from five families [7], all of them carried compound heterozygous variants, four of them were classified in the “severe phenotype” group.

**Table 4:** Recurrent nucleotide variants.

Recurrent Pc	Nucleotide variant	Authors	Cases	Associated Pc—Ho
p.R79C	c.235C>T	Yi et al.	2	p.R104C
			10	p.V314L
			11	p.V314L

p.V158M	c.472G>A	Peng et al.	11	p.I213F
			14	p.K280 ^∗^
		Wei et al.	5	p.R193C

p.R193H	c.578G>A	Slone et al.	2	p.S316Tfs ^∗^19
		Yi et al.	1	Ho
		Kaler and Couser	1	p. R277C

p.R306C	c.916C>T	Paul et al.	1, 2, 3, 4	Ho
			5	p.Q419 ^∗^
		Wei et al.	4	c.1002+1G>A
		Campbell et al.	6	Ho
		Current report	2, 3, 4	Ho

p.R309T	c.925C>T	Campbell et al.	3	p.P403L
			4	p.P403L

p.V314L	c.940G>T	Song et al.	1	p.D368N
		Wei et al.	2	c.394‐11T>G
			3	p.L351Pfs ^∗^12
		Yi et al.	5	p.R228W
			10	p.R79C
			11	p.R79C
		Kim et al.	1	Ho
		Cao et al.	2	p.W160L
		Wang et al.	1	p.R309 ^∗^

p.R315 ^∗^	c. 925C>T	Peng et al.	12	p.P409L
		Jurkute et al.	2	p.P372H
			3	p.P372H

p.C353Y	c.1058 G>A	Peng et al.	7	p.I143F
		Stenton et al.	5	p.R228L

p.D368N	c.1102G>A	Peng et al.	9, 10	Ho
		Song et al.	1	p.V314L
		Campbell et al.	7	p.M1V
		Current report	5	Ho

p.P372H	c.1115C>A	Jurkute et al.	1	p.R193C
			2, 3	p.R315 ^∗^
			5	p.A427P
			7	chr17del ^∗^
			8, 9	p.G397S

p.R386W	c.1156 C>T	Peng et al.	1, 2	Ho
			4	Ho
			5	Ho
			6	p.P74L
			13	Ho
			15	Ho
		Stenton et al.	7	Ho
		Campbell et al.	1	Ho
			2	p.P403L
			8	Ho
			9	c.270+5 G>T
			10	Ho
			13	p.G397S

p.P403L	c.1208C>T	Peng et al.	12	p.R315 ^∗^
		Campbell et al.	2	p.R386W
			3	p.R309T
			4	p.R309T

*Note:*Protein changes are displayed with the corresponded nucleotide variant. Patients displayed in the same row correspond to siblings. When not specified, patients from the same report were considered as unrelated. Asterisks are conventionally used to indicate a truncated protein resulting from a stop codon or deletion.

Abbreviations: chr17del ^∗^, chr17: 74818633—74888183del; Ho, homozygous; Pc, protein change.

Few patients carried intronic variants or deletions. Exhaustive analysis could not be performed because of the limited number of cases. However, it is worthwhile to look at the corresponding phenotype:

- Intronic variant associated with missense variant was observed in four patients (Patients 2 and 4 from Wei et al.′s study, Patients 9 and 11 from Campbell et al.′s study). All cases presented with severe phenotype. Each intronic variant was considered at high risk (≥ 98%) of consensus splice site alterations according to SpliceAI‐visual tool interpretation [22]. Similarly, one patient presented with a predicted deletion of four amino acids (p.L189_A192del, Stenton et al.′s Patient 2). As no RNA sequencing has been done, we could not include these patients in the previous domain analysis.
- One large chromosomic deletion has also been reported in one severe case (chr17: 74818633—74888183del from Jukurte et al.′s study), also excluded from the analysis as impossible to classify.

## 4. Discussion

### 4.1. Audiovestibular Disorders

HL has been reported in 38 cases so far, including those described in the current report [1, 7–9, 11, 12, 15, 17, 18, 20], with ANSD specified in 22 patients (confirmed by valid OAE responses associated with the absence of synchronized ABR signals, or expressly reported as “ANSD” or “from retrocochlear origin”) [1, 12, 15, 17, 20]. Functional hearing assessment was not detailed in the remaining cases, but one could assume that they all correspond to auditory nerve dysfunction considering FDXR involvement in mitochondria, and the ON or PNS impairments with which they are often associated.

HL appears to be more common in the milder phenotype. In addition, in children presenting with HL, the initial onset of symptoms tends to occur at an older average age onset as compared with the cohort generally: distribution of age of onset groups was as follows: 44%, 46%, and 10%, respectively for the early infancy, childhood/adolescence, and adulthood periods, whereas the distribution observed in the whole cohort was 63%, 29%, and 8%.

Considering the high proportion of optic neuropathy/atrophy consequent to this mitochondriopathy, we would expect ANSD to be as frequent as ON impairment, contrary to what was revealed this literature review (43% vs. 93%). Many hypotheses may explain this observation:

- As most patients have been followed beginning in their first years of life, HL may not yet be diagnosed and could appear later, just as described in Patient 1, who suffered from OA with severe vision loss several years before complaining of HL.
- The auditory nerve may withstand this mitochondrial default better than the ON, possibly depending on the variant pathogenicity.
- ANSD is difficult to diagnose and could be missed because PTA level may remain normal at the onset, whereas speech understanding begins to decrease.
- Hearing may be less frequently assessed, particularly in infants with severe phenotype, with CNS symptoms prioritized by physicians, resulting in HL being underdiagnosed during primary care, and consequently less reported.

Not only could delayed language or cognitive development be because of HL but also vestibular dysfunction may participate in gait disabilities in addition to the other components of balance impairment. Vestibular hyporeflexia has never been mentioned before this report, likely being underdiagnosed because of the multifactorial causes of balance disorders. Inner ear function should be systematically assessed. Nystagmus is frequent and often considered an OPH sign, but it may result from CNS or vestibular anomalies, requiring a multidisciplinary assessment.

Results of auditory rehabilitation are known to be heterogeneous in ANSD, as outcome of cochlear implantation depends on the underlying mechanism. Mitochondrial conditions induce ATP‐energy defects and consequently axonal conduction default, for which local CI stimulations cannot sufficiently compensate in most cases. In our Patients 1, 2, and 5, we assumed that CI has initially provided satisfactory results in the early course of the disease, and the later deterioration of speech intelligibility might be explained by successive flare‐ups. In our experience, long‐term prognosis of cochlear implantation appears poor. Despite satisfactory results in the first postoperative year, Patients 1, 2, and 5 presented with progressive hearing deterioration in the following year with poor comprehension.

On the other hand, good outcomes have been reported by Kim et al. in one female adult patient who underwent cochlear implantation [17]. Contrary to our patients, left cochlear nerve hypoplasia had been diagnosed, and cochlear implantation was performed on this side. Authors reported positive outcomes. However, residual hearing remained in the right ear when cochlear implantation was performed, contrary to our patients in whom surgery was decided because of the severe bilateral speech intelligibility impairments. Moreover, it would be relevant to observe CI outcomes and contralateral hearing.

It would be interesting to observe how CI outcome and the contralateral hearing would maintain over the following years considering the hearing evolution in our experience.

Consequently, information regarding the difficulty of predicting CI outcomes should be provided during preoperative counseling, keeping in mind that FDXR‐related ANSD corresponds to a postsynaptic form of ANSD, for which long‐term outcomes following CI are limited, in contrast to presynaptic ANSD, such as that observed in patients carrying pathogenic variants in OTOF and DIAPH3 [23]. Immediate postoperative outcomes may depend on the course of the disease at the time of cochlear implantation, and the benefits of CI may be greater in certain phenotypes, as described previously by Kim et al. Setting realistic expectations is especially important in these patients, who often present with multiple disabilities, particularly poor vision.

### 4.2. Literature Review

In 2017, we reported eight cases presenting with OA (more or less associated with RD) and ANSD related to *FDXR* biallelic pathogenic variations. Symptoms mostly occurred in adolescence or young adulthood. Since then, many authors reported various phenotypes. All patients described in our first report probably corresponded to “milder” phenotypes, mainly impaired by sensory disabilities (OMIM: 617717), as compared with the following publications. For instance, Peng et al. described 17 cases among 13 nonconsanguineous families [8], all of whom were symptomatic at birth or early in infancy. They presented with OA and peripheral or central nervous system symptoms (microcephaly, hypotonia, global development delay/regression or ataxia); six of them died because of severe neurologic, respiratory or feeding troubles, hence expanding FDXR disease spectrum with severe phenotypes (OMIM: 620887, MMDS9B). On the other hand, genital abnormalities have been also reported, such as ambiguous genitalia or urogenital sinus, which seem to be related to hormonal synthesis reduction [5, 10]. It is known that FDX acts as cytochrome P450 enzyme cofactor in adrenal cells [3, 6, 18] in which FDX depends on FDXR electrons transfers to be able to reduce cytochrome P450 enzymes.

Thus, it appeared interesting to bring an overview about this specific mitochondria disease′s spectrum, as genotype–phenotype correlations seem to arise from our analysis.

Firstly, it was noticeable that no biallelic truncating variant was noted, likely indicating the lethality of such genotype and consequently the critical role of FDXR in mitochondria. Our preliminary analysis also suggests that patients carrying a truncating variant exhibit a phenotype severity similar to that of patients with biallelic missense variants, depending on the associated missense variant.

In spite of the absence of functional studies, similar observation was done for intronic variants with assumption of haploinsufficiency regarding the high probability of splicing‐site disturbances.

*FDXR* pathogenic variants led to predicted Pc predominantly located within the FAD binding‐domain, suggesting that they would be less damaging to protein function. This is underscored by the higher proportion of FAD group patients among those presenting with milder phenotype. In addition, the proportion of patients with predicted Pc in FAD binding domain was higher among those with later symptom onset. On the other hand, Pc in NADPH1 and NADPH2 domains was related to severe phenotype, even more in the NADPH2 group, consistent with the fact that CNS impairment was more prevalent in patients carrying Pc exclusively located in these binding‐domains. However, the association of Pc in one NADPH binding domain with Pc in FAD domain seems to “alleviate” the phenotype with lower proportion of CNS abnormalities.

This brings up the possibility that Pc in NADPH are more severe, consistent with the current knowledge about flavoproteins, and more specifically about FDXR–FDX interactions: one pair of electrons, originated from NADPH oxidation, is captured by the FDXR NADPH binding domains and released toward FDX allowing its iron‐sulfur group reduction [6]. It potentially makes Pc in NADPH binding domains more impactful to the electron transfer.

Similarly, Pc in MTS was observed in only three patients, also suggesting a high impact on FDXR function. Moreover, all three patients′ phenotype was classified as severe. One could hypothesize that anomalies located in this domain influence the addressing signals for a proper transfer to mitochondria.

Apart from binding‐domain analysis, other observations also deserved particular attention:

- Few predicted Pc were recurrently reported; they seem to be concentrated in a specific portion at the junction between FAD and NADPH2 binding domains: nine out of 12 predicted Pc were located between the 306 and 403 amino acids positions, supporting the assumption of lower impact on FDXR function.
- Pc reported so far are mostly located away from the catalytic sites, also suggesting that they would be highly damaging, except for the 228 amino acid change observed in two unrelated patients by Stenton et al. (p.R228L) [11], and one patient by Yi et al. (p.R228W) [16]. Both authors described different phenotypes: Stenton′s patients presented with severe symptoms (microcephaly, developmental regression and psychiatric disorder in childhood), whereas Yi′s patient presented with milder phenotype (OA and retinal degeneration during the adolescence). This could be explained either by the resulting Pc (L vs. W), or by the fact that each of them carries compound heterozygous variants assuming compensatory effects of the associated variants.

All these observations bring an interesting overview about how Pc position could correlate with phenotype. However, analyzing how these Pc impact in vivo the mitochondrial function would be even more interesting. As FDXR has a critical role in the synthesis of ISPs, one would expect consequences for MRC activity, of which few ISPs are constituent parts. This has been shown in our previous work in which Complexes I/III/IV/V activities were decreased in patients cultured fibroblasts [1]. Similar results have been observed in the works of Peng and Yang, showing significant decrease of Complexes II/III and I/V activities, respectively, in their studies [8, 20]. On the other hand, cellular iron homeostasis relies on ISPs acting by posttranscriptional regulation of iron metabolism actors such as transferrin‐receptor 1. Our previous work demonstrated iron overload in patients′ fibroblasts with inconsistent Tfr1 expression increase, associated with higher cellular oxidative stress evidenced by the amount of superoxide dismutase (SOD), which has also been shown in Peng et al.′s works. Slone et al. also showed iron overload in patient fibroblasts as well as in mutant mouse cells, rescued by wild‐type FDXR expression [10].

In spite of consistent results, phenotypes of our patients were very different to those described by other teams: all patients reported by Slone, Peng, and Yang presented with severe phenotype, with early‐onset symptoms, CNS impairment, and global developmental delay in most of them, whereas our patients mostly suffered from sensorial symptoms appearing in adolescence or adulthood. It may be explained by different degrees of MRCs dysfunction or iron dysregulation depending on each variant impact. Unfortunately, functional studies are difficult to compare given differences in methodology. Further work would be then needed to understand the differential metabolic consequences between *FDXR* pathogenic variants.

We are aware that this work encounters some limitations, mainly because of the heterogeneous clinical presentation of this disease leading to differences in clinical assessment. Moreover, some anomalies may be undiagnosed at the time of genetic diagnosis because they remain asymptomatic. In addition, differences in timeline follow‐up make phenotype comparisons difficult between adults and children. In the latter, symptoms could appear later in their life. Age onset could differ depending on the reported age at the first medical visit. We keep in mind that phenotype severity relied on our judgment, but they were considered as severe when obvious, especially in patients presenting with central neurological development delay.

## 5. Conclusion

In conclusion, much remains to be understood about this mitochondrial disease. Progressive HL with early impact on speech intelligibility is frequently observed. CI may be helpful at some point in disease progression, keeping in mind that outcomes may vary according to disease stage or genotype. Long‐term follow‐up is necessary, and particular attention should be paid not only to the neurologic or neurosensorial symptoms, but also to the potential hormonal dysfunction considering FDXR′s involvement in both ISP and steroid hormone synthesis. It is hoped that this work will raise physicians′ awareness of systematic multidisciplinary evaluation with long term follow‐up that would contribute to our future understanding of FDXR disease.

Despite our analysis supporting the correlation between Pc and phenotype severity, molecular studies must still be performed to understand how Pc observed in patients may influence FDXR‐FDX interaction and how it would impact mitochondrial function and iron regulation. As FDXR and frataxin proteins share a common metabolic pathway of ISP synthesis, it is hoped that ongoing works in FA disease, studied for much longer than FDXR disease, may lead to therapy candidates that could treat both conditions.

## Funding

No funding was received for this manuscript.

## Conflicts of Interest

The authors declare no conflicts of interest.

## Supporting Information

Additional supporting information can be found online in the Supporting Information section.

## Supporting information


**Supporting Information 1** Figure S1: Patient 1. (A) Preoperative assessment. A.1: PTA and SRT, grey: first visit, colored: preoperative PTA and SRT. A.2: OAE and ABR, validated OAE responses in both ears, no identifiable ABR. A.3: Caloric tests revealing bilateral severe hyporeflexia with low angular velocity and mild left preponderance. (B) Postoperative results: PTA and SRT. B.1: Audiometry at the 8th postoperative month after right cochlear implantation. B.2: Audiometry at the 18th postoperative month after right cochlear implantation. B.3: Audiometry at one postoperative year after left cochlear implantation. PTA: Pure tone audiometry, SRT: speech recognition scores, OAE: otoacoustic emissions, ABR: auditory brainstem responses, CI: cochlear implant, HA: hearing aid, wo: without.

**Supporting Information 2** Figure S2: Patient 2. (A) PTA and speech recognition scores at the first visit and 2 years later. (B) Transient otoacoustic emissions (present) and auditory brainstem responses (unsynchronized shaped). (C) Vestibular caloric tests showing bilateral hyporeflexia with low angular velocity < 10/s. (D) Postoperative audiometry.

**Supporting Information 3** Figure S3: Family 2—Patient 3. (A) PTA and speech recognition scores at the first visit and 3 years later. (B) Valid transient otoacoustic emissions and auditory brainstem responses (low amplitude with hardly reproducible signals).

**Supporting Information 4** Figure S4: Patient 4. (A) PTA and speech recognition scores at the first/second visits and 4 years later. (B) Transient otoacoustic emissions (completely present in the right ear, validated only at 2 kHz in the left ear) and auditory brainstem responses (shape of unsynchronous signals in both ears).

**Supporting Information 5** Figure S5: Patient 5. (A) Preoperative audiometry—pure tone average and speech recognition scores. (B) Preoperative auditory brainstem responses. (C) Postoperative audiometry results: 6 months after right cochlear implantation; 6 years after bilateral implantation. CI: cochlear implant.

**Supporting Information 6** Table S1: Detailed phenotypes of patients reported up to March 2025. Clinical signs were categorized as follows: central or peripheral nervous system (CNS, PNS), ophthalmological signs (OPH), hearing loss (HL) and others. Each case number corresponds to that indicated in Table 2.

**Supporting Information 7** Table S2: Mild and severe phenotypes among patients carrying either biallelic missense variants in one same binding‐domain, and those with truncating associated with missense variants. Patients carrying biallelic missense variants analyzed in this table were those classified in the NADPH1, FAD, and NADPH2 groups, for a consistent comparison with the missense variant of the other group. Patients carrying truncating variants were those described by Paul (Patient 5), Peng (Patients 12 and 14), Jurkute (one of the two siblings, Patients 2 and 3), Campbell (Patients 5, 12, and 14), Wang (Patient 1), Cao (Patient 1), Slone (Patient 2), and Wei (Patients 1 and 3). In both groups, variants in FAD domain seemed to be more related to mild phenotypes, contrary to those in NADPH2.

## Data Availability

The data that supports the findings of this study are available in the Supporting Information of this article.

## References

[1] Paul A. , Drecourt A. , Petit F. , Deguine D. D. , Vasnier C. , Oufadem M. , Masson C. , Bonnet C. , Masmoudi S. , Mosnier I. , Mahieu L. , Bouccara D. , Kaplan J. , Challe G. , Domange C. , Mochel F. , Sterkers O. , Gerber S. , Nitschke P. , Bole-Feysot C. , Jonard L. , Gherbi S. , Mercati O. , Ben Aissa I. , Lyonnet S. , Rötig A. , Delahodde A. , and Marlin S. , FDXR Mutations Cause Sensorial Neuropathies and Expand the Spectrum of Mitochondrial Fe-S-Synthesis Diseases, American Journal of Human Genetics. (2017) 101, no. 4, 630–637, 10.1016/j.ajhg.2017.09.007, 28965846. PMC5630197 28965846

[2] Stehling O. , Wilbrecht C. , and Lill R. , Mitochondrial Iron-Sulfur Protein Biogenesis and Human Disease, Biochimie. (2014) 100, 61–77, 10.1016/j.biochi.2014.01.010. 24462711

[3] Grinberg A. V. , Hannemann F. , Schiffler B. , Müller J. , Heinemann U. , and Bernhardt R. , Adrenodoxin: Structure, Stability, and Electron Transfer Properties, Proteins: Structure, Function, and Bioinformatics. (2000) 40, no. 4, 590–612, 10.1002/1097-0134(20000901)40:4<590::AID-PROT50>3.0.CO;2-P, 10899784. 10899784

[4] Vickery L. E. , Molecular Recognition and Electron Transfer in Mitochondrial Steroid Hydroxylase Systems, Steroids. (1997) 62, no. 1, 124–127, 10.1016/S0039-128X(96)00170-5, 9029726. 9029726

[5] Pignatti E. , Slone J. , Gómez Cano M. Á. , Campbell T. M. , Vu J. , Sauter K. S. , Pandey A. V. , Martínez-Azorín F. , Alonso-Riaño M. , Neilson D. E. , Longo N. , du Toit T. , Voegel C. D. , Huang T. , and Flück C. E. , FDXR variants Cause Adrenal Insufficiency and Atypical Sexual Development, JCI Insight. (2024) 9, no. 14, e179071, 10.1172/jci.insight.179071, 38885337. PMC11383170 38885337

[6] Miller W. L. , Steroid Hormone Synthesis in Mitochondria, Molecular and Cellular Endocrinology. (2013) 379, no. 1–2, 62–73, 10.1016/j.mce.2013.04.014. 23628605

[7] Jurkute N. , Shanmugarajah P. D. , Hadjivassiliou M. , Higgs J. , Vojcic M. , Horrocks I. , Nadjar Y. , Touitou V. , Lenaers G. , Poh R. , Acheson J. , Robson A. G. , Raymond F. L. , Reilly M. M. , Yu-Wai-Man P. , Moore A. T. , Webster A. R. , Arno G. , and for the Genomics England Research Consortium , Expanding the *FDXR*-Associated Disease Phenotype: Retinal Dystrophy Is a Recurrent Ocular Feature, Investigative Ophthalmology & Visual Science. (2021) 62, no. 6, 10.1167/iovs.62.6.2, 33938912. PMC8107637 33938912

[8] Peng Y. , Shinde D. N. , Alexander Valencia C. , Mo J. S. , Rosenfeld J. , Cho M. T. , Chamberlin A. , Li Z. , Liu J. , and Gui B. , Biallelic Mutations in the Ferredoxin Reductase Gene Cause Novel Mitochondriopathy With Optic Atrophy, Human Molecular Genetics. (2018) 27, no. 12, 10.1093/hmg/ddy072, 29554255. PMC5985726 29554255

[9] Wei X. , Li H. , Zhu T. , Yao F. , and Sui R. , FDXR-Associated Disease in a Chinese Cohort: Unraveling Expanded Ocular Phenotypes and Genetic Spectrum, Experimental Eye Research. (2023) 234, 109600, 10.1016/j.exer.2023.109600, 37481223. 37481223

[10] Slone J. , Peng Y. , Chamberlin A. , Harris B. , Kaylor J. , McDonald M. T. , Lemmon M. , el-Dairi M. A. , Tchapyjnikov D. , Gonzalez-Krellwitz L. A. , Sellars E. A. , McConkie-Rosell A. , Reinholdt L. G. , and Huang T. , Biallelic Mutations in FDXR Cause Neurodegeneration Associated With Inflammation, Journal of Human Genetics. (2018) 63, no. 12, 1211–1222, 10.1038/s10038-018-0515-y, 30250212. PMC6451867 30250212

[11] Stenton S. L. , Piekutowska-Abramczuk D. , Kulterer L. , Kopajtich R. , Claeys K. G. , Ciara E. , Eisen J. , Płoski R. , Pronicka E. , Malczyk K. , Wagner M. , Wortmann S. B. , and Prokisch H. , Expanding the Clinical and Genetic spectrum of FDXR Deficiency by Functional Validation of Variants of Uncertain Significance, Human Mutation. (2021) 42, no. 3, 310–319, 10.1002/humu.24160, 33348459. 33348459

[12] Yang C. , Zhang Y. , Li J. , Song Z. , Yi Z. , Li F. , Xue J. , Zhang W. , and Wang C. , Report of a Case With Ferredoxin Reductase (*FDXR*) Gene Variants in a CHINESE BOY EXHIBITING Hearing Loss, Visual Impairment, and Motor Retardation, International Journal of Developmental Neuroscience. (2021) 81, no. 4, 364–369, 10.1002/jdn.10104, 33742450. 33742450

[13] Keller N. , Paketci C. , Altmueller J. , Fuhrmann N. , Wunderlich G. , Schrank B. , Unver O. , Yilmaz S. , Boostani R. , Karimiani E. G. , Motameny S. , Thiele H. , Nürnberg P. , Maroofian R. , Yis U. , Wirth B. , and Karakaya M. , Genomic Variants Causing Mitochondrial Dysfunction Are Common in Hereditary Lower Motor Neuron Disease, Human Mutation. (2021) 42, no. 4, 460–472, 10.1002/humu.24181, 33600046. 33600046

[14] Song S. J. , Hong Y. , Xu K. , and Zhang C. , Novel Biallelic Compound Heterozygous Mutations in FDXR Cause Optic Atrophy in a Young Female Patient: A Case Report, International Journal of Ophthalmology. (2021) 14, no. 11, 1796–1798, 10.18240/ijo.2021.11.22, 34804873. PMC8569559 34804873

[15] Masnada S. , Previtali R. , Erba P. , Beretta E. , Camporesi A. , Chiapparini L. , Doneda C. , Iascone M. , Sartorio M. U. A. , Spaccini L. , Veggiotti P. , Osio M. , Tonduti D. , and Moroni I. , FDXR-Associated Disease: A Challenging Differential Diagnosis With Inflammatory Peripheral Neuropathy, Neurological Sciences. (2023) 44, no. 9, 3037–3043, 10.1007/s10072-023-06790-0, 37046037. PMC10096094 37046037

[16] Yi S. , Zheng Y. , Yi Z. , Wang Y. , Jiang Y. , Ouyang J. , Li S. , Xiao X. , Sun W. , Wang P. , and Zhang Q. , FDXR-Associated Oculopathy: Congenital Amaurosis and Early-Onset Severe Retinal Dystrophy as Common Presenting Features in a Chinese Population, Genes. (2023) 14, no. 4, 10.3390/genes14040952, 37107710. PMC10137360 37107710

[17] Kim B. J. , Kim Y. , Kim J. A. , Han J. H. , Kim M. Y. , Yang H. K. , Rhee C. S. , Kang Y. C. , Kim C. H. , and Choi B. Y. , Novel Variant of FDXR as a Molecular Etiology of Postlingual Post-Synaptic Auditory Neuropathy Spectrum Disorder via Mitochondrial Dysfunction: Reiteration of the Correlation Between Genotype and Cochlear Implantation Outcomes, Clinical and Experimental Otorhinolaryngology. (2024) 17, no. 3, 206–216, 10.21053/ceo.2024.00184, 39104018. PMC11375174 39104018

[18] Campbell T. , Slone J. , Metzger H. , Liu W. , Sacharow S. , Yang A. , Moosajee M. , la Morgia C. , Carelli V. , Palombo F. , Lines M. A. , Innes A. M. , Levy R. J. , Neilson D. , Longo N. , and Huang T. , Clinical Study of Ferredoxin-Reductase-Related Mitochondriopathy: Genotype-Phenotype Correlation and Proposal of Ancestry-Based Carrier Screening in the Mexican population, Genetics in Medicine Open. (2024) 2, 100841, 10.1016/j.gimo.2023.100841, 39669623. PMC11613914 39669623

[19] Kaler A. and Couser N. , Ocular and Neurological Manifestations of the FDXR-Related Disorder, Journal of the American Association for Pediatric Ophthalmology and Strabismus. (2025) 29, no. 1, 104102, 10.1016/j.jaapos.2024.104102, 39746537. 39746537

[20] Cao Y. , Zhang X. , Lan L. , Li D. , Li J. , Xie L. , Xiong F. , Yu L. , Wu X. , Wang H. , and Wang Q. , Identification of Genetic Mechanisms of Non-Isolated Auditory Neuropathy With Various Phenotypes in Chinese Families, Orphanet Journal of Rare Diseases. (2025) 20, no. 1, 10.1186/s13023-025-03540-7, 39780253. PMC11715445 39780253

[21] Wang J. , Zhao R. , Ma J. , Qin J. , Zhang H. , Guo J. , Chang X. , and Zhang W. , Biallelic FDXR Mutations Induce Ferroptosis in a Rare Mitochondrial Disease With Ataxia, Free Radical Biology and Medicine. (2025) 230, 248–262, 10.1016/j.freeradbiomed.2025.02.012, 39954867. 39954867

[22] De Sainte Agathe J.-M. , Filser M. , Isidor B. , Besnard T. , Gueguen P. , Perrin A. , Van Goethem C. , Verebi C. , Masingue M. , Rendu J. , and Cossée M. , SpliceAI-visual: A Free Online Tool to Improve SpliceAI Splicing Variant Interpretation, Human Genomics. (2023) 17, no. 1, 10.1186/s40246-023-00451-1, 36765386. PMC9912651 36765386

[23] Rance G. and Starr A. , Pathophysiological Mechanisms and Functional Hearing Consequences of Auditory Neuropathy, Brain. (2015) 138, no. 11, 3141–3158, 10.1093/brain/awv270, 26463676. 26463676

