# Supplementary material for: Genotype–Phenotype Relationships in Patients Carrying Biallelic FDXR Pathogenic Variants—New Cases and Systematic Review
Source: Hum Mutat. 2026 Oct 2;2026:5928629. doi: 10.1155/humu/5928629 (PMC13633460; doi:10.1155/humu/5928629)
Supplement: Supplementary file 1 [file HUMU-2026-5928629-s001.docx]

**A**

**A.3**

**A.1**

**A.2**

**B**

Speech recognition scores

A-A: free-field, right CI, no controlateral hearing aid S-S: free-field, right CI + controlateral hearing aid

Left ear
wo HA

Right CI

Speech recognition scores
C-C: free-field, right CI, no controlateral hearing aid

**B.2**

**B.1**

Speech recognition scores
A-A: bilateral CI, A-A: right CI, A2-A2: left CI

**B.3**

**Figure S1: patient 1
A. Pre-operative assessment**
A.1: PTA (left) and SRT (right), Grey: first visit, Coloured: pre-operative PTA and SRT
A.2: OAE (left) and ABR (right), validated OAE responses in both ears, no identifiable ABR
A.3: Caloric tests revealing bilateral severe hyporeflexia with low angular velocity and mild left preponderance **B. Post-operative results: PTA(left) and SRT(right)**
B.1: Audiometry at the 8th post-operative month after right cochlear implantation
B.2: Audiometry at the 18th post-operative month after right cochlear implantation
B.3: Audiometry at one post-operative year after left cochlear implantation
PTA: Pure tone audiometry, SRT: Speech recognition scores, OAE: otoacoustic emissions, ABR: auditory brainstem responses, CI: cochlear implant, HA: hearing aid, wo: without.

**Figure S1: patient 1
A. Pre-operative assessment**
A.1: PTA (left) and SRT (right), Grey: first visit, Coloured: pre-operative PTA and SRT
A.2: OAE (left) and ABR (right), validated OAE responses in both ears, no identifiable ABR
A.3: Caloric tests revealing bilateral severe hyporeflexia with low angular velocity and mild left preponderance **B. Post-operative results: PTA(left) and SRT(right)**
B.1: Audiometry at the 8th post-operative month after right cochlear implantation
B.2: Audiometry at the 18th post-operative month after right cochlear implantation
B.3: Audiometry at one post-operative year after left cochlear implantation
PTA: Pure tone audiometry, SRT: Speech recognition scores, OAE: otoacoustic emissions, ABR: auditory brainstem responses, CI: cochlear implant, HA: hearing aid, wo: without.

Right ear
wo CI

Right ear
wo CI

Left ear
wo HA

A2-A2 bilateral CI
