# Supplementary material for: Genotype–Phenotype Relationships in Patients Carrying Biallelic FDXR Pathogenic Variants—New Cases and Systematic Review
Source: Hum Mutat. 2026 Oct 2;2026:5928629. doi: 10.1155/humu/5928629 (PMC13633460; doi:10.1155/humu/5928629)
Supplement: Supplementary file 2 [file HUMU-2026-5928629-s002.docx]

**A**

Two years later
Green/black lines: PTA and SRT with bilateral hearing aid
Red/Blue: right/left hearing with(w) and without(wo) hearing aid

w

wo

w

wo

w

wo

w

wo

First audiometry

TEOAE

ABR

**B**

**C**

2 years post-operative evaluation
Red: right ear with hearing aid only, Blue: square = left ear with cochlear implant only, Cross = left ear without cochlear implant.

**D**

**Figure S2: patient 2**
A: PTA and speech recognition scores at the first visit and two years later
B: transient otoacoustic emissions (present) and auditory brainstem reponses (unsynchronized shaped)
C: vestibular caloric tests showing bilateral hyporeflexia with low angular velocity < 10°/s
D. post-operative audiometry
