# Supplementary material for: Genotype–Phenotype Relationships in Patients Carrying Biallelic FDXR Pathogenic Variants—New Cases and Systematic Review
Source: Hum Mutat. 2026 Oct 2;2026:5928629. doi: 10.1155/humu/5928629 (PMC13633460; doi:10.1155/humu/5928629)
Supplement: Supplementary file 3 [file HUMU-2026-5928629-s003.docx]

**Figure S3: family 2 – patient 3**
A: PTA and speech recognition scores at the first visit and three years later
B: valid transient otoacoustic emissions and auditory brainstem responses (low amplitude with hardly reproducible signals)

**Figure S3: family 2 – patient 3**
A: PTA and speech recognition scores at the first visit and three years later
B: valid transient otoacoustic emissions and auditory brainstem responses (low amplitude with hardly reproducible signals)

**B**

**A**

ABR

TEOAE

Left

Right

Left ear

Right ear

Three years later

First audiometry
