# Supplementary material for: Genotype–Phenotype Relationships in Patients Carrying Biallelic FDXR Pathogenic Variants—New Cases and Systematic Review
Source: Hum Mutat. 2026 Oct 2;2026:5928629. doi: 10.1155/humu/5928629 (PMC13633460; doi:10.1155/humu/5928629)
Supplement: Supplementary file 4 [file HUMU-2026-5928629-s004.docx]

**Figure S4: patient 4**
A: PTA and speech recognition scores at the first/second visits and four years later
B: B.1: transient otoacoustic emissions (completely present on the right ear, validated only at 2kHz on the left ear), B.2: auditory brainstem responses (shape of unsynchronous signals in both ears)

**Figure S4: patient 4**
A: PTA and speech recognition scores at the first/second visits and four years later
B: B.1: transient otoacoustic emissions (completely present on the right ear, validated only at 2kHz on the left ear) auditory brainstem responses (shape of unsynchronous signals in both ears)

**A**

Audiometry at the first visit (dotted line) and two years later (solid line)

Right ear

Left ear

Four years later

TEOAE

ABR

**B**

B.1

B.2
