# Supplementary material for: Genotype–Phenotype Relationships in Patients Carrying Biallelic FDXR Pathogenic Variants—New Cases and Systematic Review
Source: Hum Mutat. 2026 Oct 2;2026:5928629. doi: 10.1155/humu/5928629 (PMC13633460; doi:10.1155/humu/5928629)
Supplement: Supplementary file 5 [file HUMU-2026-5928629-s005.docx]

**Figure S5: patient 5**
A: pre-operative audiometry – Pure tone average and speech recognition scores
B: preoperative auditory brainstem responses
C: post-operative audiometry results.
 Left: six months after right cochlear implantation, Right: six year after bilateral implantationµ
CI: cochlear implant

**C**

Sixth post-operative month (first CI)

A-A: right CI results

Sixth post-operative year

Red: right CI, Blue: left CI, Black: bilateral CI

Pre-operative ABR

**B**

Right ear

Left ear

**A**

Right ear

Left ear

Speech recognition scores

dB

%

dB
