# Supplementary material for: Genotype–Phenotype Relationships in Patients Carrying Biallelic FDXR Pathogenic Variants—New Cases and Systematic Review
Source: Hum Mutat. 2026 Oct 2;2026:5928629. doi: 10.1155/humu/5928629 (PMC13633460; doi:10.1155/humu/5928629)
Supplement: Supplementary file 6 [file HUMU-2026-5928629-s006.docx]

| **Authors** | **Cases** | **Phenotype** |
| --- | --- | --- |
| **Paul *et al.***  **2017** | **1** | - HL: auditory neuropathy spectrum disorder - OPH: optic atrophy |
|  | **2** | - HL: auditory neuropathy spectrum disorder - OPH: optic atrophy |
|  | **3** | - HL: auditory neuropathy spectrum disorder - OPH: optic atrophy |
|  | **4** | - HL: auditory neuropathy spectrum disorder - OPH: optic atrophy |
|  | **5** | - HL: auditory neuropathy spectrum disorder - OPH: optic atrophy |
|  | **6** | - HL: auditory neuropathy spectrum disorder - OPH: retinal dystrophy, optic atrophy - PNS: hypopallesthesia |
|  | **7** | - HL: auditory neuropathy spectrum disorder - OPH: optic atrophy |
|  | **8** | - HL: auditory neuropathy spectrum disorder - OPH: optic atrophy |
| **Peng *et al.***  **2017** | **1** | - CNS: hypotonia, global developmental delays - OPH: small optic nerves, delayed myelination, bilateral optic nerve pallor, cherry red spots, poor visual tracking |
|  | **2** | - CNS: motor development, hypotonia, cortical atrophy - OPH: bilateral optic nerve pallor, cherry red spots, poor visual tracking, strabismus - Others: Premature death (17 months), chronic respiratory failure, lost his oral feeding skills |
|  | **3** | - CNS: spastic gait - PNS: axonal sensorimotor polyneuropathy of the lower extremities, absent joint position sense in the lower extremities, absent vibratory sensation (lower limbs) - OPH: blindness, night vision difficulties, decrease in visual field, delayed rod response, bilateral retinitis pigmentosa, beats of horizontal clonus - Others: high pitched voice, bilateral spastic planovalgus foot deformities, valgus foot deformities, knee valgus, hyperextensible elbows, tight hamstrings bilaterally with bilateral hallux valgus, wide intercondyle distance |
|  | **4** | - CNS: microcephaly, developmental delays, failure to thrive, decreased muscle strength and tone, cortical vision impairment, involuntary hand movement - PNS: mild joint contractures - Others: Early death (6years), Neonatal severe respiratory failure, Growth defect: weight, length, head circumference below the 3rd percentile |
|  | **5** | - CNS: Poor head control, hypotonia and consequent swallowing difficulties, global developmental delay, strabismus, mild cerebellar volume loss, thin corpus callosum, chronic extra-axial collections in bilateral frontal convexities, diffuse disturbance in cerebral function on EEG - OPH: optic atrophy, poor fixation and optic nerve pallor for both eyes - HL: abnormal auditory brainstem responses - Others: failure to thrive |
|  | **6** | - CNS: microcephaly, developmental delays, hypoventilation, movement disorder cerebellar, hyperkinetic movements, dystonic movements/agitation, , cortical visual impairment, difficulty swallowing - PNS: muscle tone abnormalities, spasticity, muscle weakness - OPH: small optic nerves (optic atrophy), optic discs with “salt/pepper appearance” - Others: Limbs/rachis affections (loose-jointed, scoliosis, sacral dimple), short statue, poor growth |
|  | **7** | - CNS: progressive encephalopathy, regression of motor milestones, quadriplegia and global developmental delay - OPH: optic atrophy |
|  | **8** | - CNS: delays in, microcephaly both gross and fine motor development, poor head control, generalized hypotonia, head circumference decreasing, seizure, abnormal EEG, - OPH: poor vision, bilateral disc pallor, “atrophy looking retina” - Other: respiratory failure (BiPAP) |
|  | **9** | - CNS: cognitive loss, encephalopathy, microcephaly, intellectual delay - PNS: motor and sensory neuropathy, pain in lower limbs, muscle weakness - OPH: vision impairment, nystagmus - HL: bilateral hearing loss |
|  | **10** | - CNS: cognitive loss, encephalopathy, feeding difficulty, microcephaly, cord with diffuse atrophy T2-T10 - PNS: pain in lower limbs, muscle weakness, contractures at knees and fingers - OPH: vision impairment, nystagmus - HL: bilateral hearing loss, |
|  | **11** | - CNS: hypotonia, speech apraxia, gross and fine motor skill delays, bilateral Babinski signs, poor balance, cognitive progressing disorder - PNS: myopathy , severe progressive axonal neuropathy affecting position sensation, fixed contractures in his feet and ankles, no position sensation in his toes or ankles, spastic quadriplegia, EMG: mixed, axon loss, sensorimotor peripheral polyneuropathy - HL: mild to moderate sensorineural hearing loss, expressive language disorder - OPH: progressive vision impairment, retinitis pigmentosa, optic nerve atrophy, photosensitivity, nyctalopia, and peripheral field constriction - Others: pes cavus deformity bilaterally of the feet |
|  | **12** | - CNS: motor delays ( walking at 22mo), language delay, ataxia - PNS: decreased muscle strength - OPH: strabismus, vision loss and night vision problems, retinopathy |
|  | **13** | - CNS: developmental delay, hypotonia, eyes rolling, stereotypical hand movements, disconjugate gaze, nonreactive pupils, delayed milestones - OPH: hypoplasia of bilateral optic nerves and optic chiasm   Other: pyloric stenosis |
|  | **14** | - CNS: abnormal gait, delayed language development, hypotonia, complete vision loss, Seizures, cerebellar atrophy, EEG: Mild diffuse slowing, diffuse slowing - PNS: lower extremity spasticity, brisk reflexes, EMG: widespread slowing of nerve conductions (motor and sensory nerves) - OPH: nystagmus, complete vision loss, bilateral optic atrophy - HL: severe to profound bilateral sensorineural hearing loss - Other: Dead at the age of 32 |
|  | **15** | - OPH: poor visual tracking, small optic nerves and pigment retinopathy bilaterally - CNS: global developmental delay and hypotonia, chronic respiratory failure, dysphagia/ abnormal swallow ability |
| **Slone *et al.***  **2018** | **1** | - CNS: Global developmental delay, delays in speech, fine motor, gross motor skills of age, ataxia, gait abnormality - OPH: poor vision, bilateral optic atrophy, scotoma, thinning of the ganglion cell layer |
|  | **2** | - CNS: poor feeding, initial respiratory distress syndrome, cerebellar vermis hypoplasia, failure to thrive, global developmental delay, and muscular hypertonicity, global cerebral atrophy - OPH: bilateral thin optic nerves, bilateral cataracts - Others: - Early death (10.5), ambiguous genitalia and clitoromegaly, urogenital sinus; immature ovarian parenchyma, clitoromegaly, labial fusion, congestive heart failure, hepatosplenomegaly, pulmonary congestion and edema(autopsy), weight, length, head circumference < 5th percentile |
| **Stenton *et al.***  **2020** | **1** | - CNS: Dystonia, ataxia, psychiatric disorder - PNS /muscular: Myopathy, hypotonia, ptosis, ophthalmoplegia, polyneuropathy - OPH: optic atrophyHL: “hearing loss” - Others: diabetes mellitus |
|  | **2** | - CNS: seizures, developmental delay, respiratory insufficiency, Leigh syndrome, blood pressure regulation - PNS: myopathy - OPH: optic atrophy, nystagmus - Other: Premature death, anemia |
|  | **3** | - CNS: Microcephaly, developmental regression, movement disorder, ataxia - HL: “hearing loss” |
|  | **4** | - CNS: Microcephaly, movement, disorder, ataxia - OPH: optic atrophy |
|  | **5** | - CNS: Microcephaly, developmental regression, progressive encephalopathy, hypotonia, respiratory distress, feeding difficulties, involuntary movement, inability to sit or to stabilize the head - PNS: limb spasticity, incomplete knee extension - OPH: optic atrophy, cataract - HL: bilateral severe hearing loss - Others: poor weight gain, delayed bone age |
|  | **6** | - CNS: Developmental regression, ataxia - PNS: spasticity - OPH: optic atrophy - HL: “hearing loss“ |
|  | **7** | - CNS: Global developmental delay, developmental, regression, non-ambulatory - PNS: hypotonia - OPH: optic atrophy strabismus |
| **Yang *et al.***  **2021** | **1** | - CNS: developmental delay, unable to lift his head stably, was unable to turnover, and his eyes did not track properly, hypotonia - OPH: optic disc of both eyes was pale, the blood vessels were thin, white lines were observed, and the surrounding vitreous body of both eyes were point-like opacity - HL: auditory neuropathy of spectrum disorder - Others: born at 36 weeks of unexplained premature delivery, Premature death at the age of 1, unexplained jaundice, septic shock |
| **Keller et al.**  **2021** | **1** | - CNS: global developmental delay syndrome - PNS: ataxia, peripheral neuropathy - OPH: Optic atrophy |
| **Song et al.**  **2021** | **1** | - OPH: optic atrophy, retinal thickness, temporal, visual field defects, centric scotoma |
| **Jukurte et al.**  **2021** | **1** | - OPH: decreased vision, convergent squint, small pale optic nerves, slender optic nerves and chiasm, bilateral retinal ganglion cell/optic nerve dysfunction, nystagmus - Others: tremor, dizziness and balance |
|  | **2** | - OPH: Visual constricted fields, bilateral optic atrophy, pigment migration in the mid periphery of the retina - HL: mild sensorineural hearing loss - PNS: peripheral neuropathy |
|  | **3** | - PNS: peripheral neuropathy - OPH: reduced vision under low light conditions, severe photoreceptor dysfunction with severe macular involvement, cataracts, bilateral optic atrophy, retinal vessel attenuation, peripheral pigmentary changes, macular edema at the age of 5 - HL: profound hearing impairment at the age of 5 - Others: verbal dyspraxia |
|  | **4** | - OPH: bilateral optic atrophy and retinal dystrophy, pale optic nerves, atrophy retina, and a slight epiretinal membrane bilaterally, retinal dysfunction, affecting both rods and cones, loss of retinal vessels, retinal pigmentary changes. early cataract bilaterally - HL: sensorineural hearing loss - Others: tremor (both arms) at the age of 17, horizontal nystagmus in both eyes at the age of 14, changing to pendular |
|  | **5** | - PNS: relapse and lower limb distal flaccid paresis with areflexia and peripheral dysesthesia. Peripheral motor and sensory neuropathy involving the lower limbs. Axonal loss, secondary to the demyelination process - OPH: severe reduced vision in both eyes at the age of 24. Atrophy pale optic nerve head bilaterally - Others: reduction in exercise tolerance |
|  | **6** | - CNS: mild cerebellar syndrome - PNS: foot pain with pins and needles, mild neuropathy, minimal weakness of ankle dorsiflexion, absent lower limb reflexes, severe axonal length dependent polyneuropathy (lower limbs) - HL: bilateral progressive mild to severe deafness - OPH: bilateral optic atrophy, bilateral demyelinating optic neuropathy - Others: intermitted psychosis |
|  | **7** | - CNS: ataxia due to poor coordination and balance - OPH: retinitis pigmentosa, bilateral nystagmus and optic atrophy, cataracts at the age of 17 - Others: attention deficit hyperactivity disorder, hypermobility. |
|  | **8** | - CNS: posterior column disease with mild gait ataxia - PNS: brisk knee jerks, absent ankle jerks, and impaired vibration sense to the knee - OPH: optic atrophy and retinal dystrophy - Others: gastrointestinal tract disorder |
|  | **9** | - CNS/PNS: “neurological symptoms”, absent ankle jerks, impaired joint position, and vibration sense and gait ataxia - OPH: optic atrophy and rod cone dystrophy |
|  | **10** | - PNS: neuropathic pain, motor neuropathy and subsequent sensory ataxia and motor weakness - OPH: optic atrophy, retinal pigmentary changes in the far periphery nasal to the disc, outer retinal thinning at the macula - HL: “hearing loss” - Others: vomiting, constipation, severe weight loss. |
| **Masnada *et al.***  **2023** | **1** | - PNS: lower limbs proximal and mainly distal asymmetric muscle weakness and reduced tendon reflexes, alterations of central conduction at lower limbs, hyperesthesia at distal upper and lower limbs - OPH: optic neuropathy at the age of 13, scotopic retinal alterations - HL: auditory impairment, dysfunction of the brainstem acoustic pathway of peripheral and retrocochlear origin - Others: enuresis, walking disturbances, difficulties in autonomous gait and frequent falls |
|  | **2** | - CNS: mild dysarthria, diffuse hypotonia - PNS: upper limb hyperreflexia and proximal weakness, lower limb areflexia and flaccid paralysis, dysesthesia, and pain in distal regions - OPH: slight chiasm and optic nerves atrophy - Others: severe abdominal pain, flaccid paralysis with loss of trunk control and autonomous deambulation |
| **Yi *et al*. 2023** | **1** | - CNS: developmental delay and could not lift her head at one year of age - OPH: severe retinopathy resembling Leber congenital amaurosis, poor visual tracking, sluggish pupillary light reflex, and undetectable ERG records, optic disc pallor, silver wiring of retinal vessels, and generalized salt-and-pepper retinal degeneration - Others: death at the age of 13 (pneumonia), nystagmus within the first few months after birth, born prematurely at 34 weeks of pregnancy, hand, foot, and mouth disease |
|  | **2** | - CNS: mild mental retardation - OPH: severe severe retinopathy resembling Leber congenital amaurosis, poor visual tracking, sluggish pupillary light reflex, poor vision at one year of age, undetectable ERG records, optic disc pallor, silver wiring of retinal vessels, and generalized salt-and-pepper retinal degeneration - Others: nystagmus within the first few months after birth, “bump into things while walking” |
|  | **3** | - CNS: microcephaly, hypertonia, and developmental delay, ataxia at two years of age, cerebellar atrophy - OPH: severe severe retinopathy resembling Leber congenital amaurosis, poor visual tracking, sluggish pupillary light reflex, esotropia, and hyperopia refractive error, undetectable ERG records, optic disc pallor, silver wiring of retinal vessels, and generalized salt-and-pepper retinal degeneration - Others: nystagmus within the first few months after birth |
|  | **4** | - OPH: severe severe retinopathy resembling Leber congenital amaurosis, poor visual tracking, sluggish pupillary light reflex, and undetectable ERG records, optic disc pallor, silver wiring of retinal vessels, and generalized salt-and-pepper retinal degeneration, thick optic nerve - Others: nystagmus within the first few months after birth, muscle weakness in the lower extremity |
|  | **5** | - OPH: nyctalopia, poor vision, early onset of retinal dystrophy, optic atrophy, silver-wiring retinal vessels   and tapetoretinal degeneration |
|  | **6** | - OPH: nyctalopia, poor vision, early onset of retinal dystrophy, optic atrophy, silver-wiring retinal vessels   and tapetoretinal degeneration |
|  | **7** | - OPH: nyctalopia, strabismus, optic disc pallor, silver wiring of retinal vessels, and salt-and-pepper retinal degeneration, retinal thinning |
|  | **8** | - OPH: vision impairment, Complete optic disc pallor, silver wiring of retinal vessels, and generalized tapetoretinal degeneration, Macular involvement, disruptions of the interdigitation and ellipsoid zones, shallow foveal pit, reduced retinal thickness. - Others: nystagmus |
|  | **9** | - OPH: nyctalopia, at the age of three, complete optic disc pallor, silver wiring of retinal vessels, and salt-and-pepper retinal degeneration |
|  | **10** | - OPH: poor vision, complete optic disc pallor, severe attenuation of retinal vessels, and tapetoretinal degeneration |
|  | **11** | - OPH: poor vision, complete optic disc pallor, severe attenuation of retinal vessels, and tapetoretinal degeneration, scotopic and photopic responses were severely reduced |
| **Wei *et al.***  **2023** | **1** | - CNS: ataxia, cerebellar atrophy - OPH: pale optic nerves, attenuation of the retinal vessels, and pepper pigmentation, disorganized retinal structure in the macula, thinning of the ganglion cell layer, discontinuity of the ellipsoid zone, and cystoid macular edema, pigmentation in the midperipheral retina, extinguished scotopic and photopic responses - HL: sensorineural hearing loss - Others: balance issues, insomnia, deterioration of her movement disorder |
|  | **2** | - CNS: seizures - OPH: visual impairment, night blindness, and photophobia, pale optic nerves, retinal degeneration with pepper pigmentation, attenuation of the retinal vessels, pigmentation of the peripheral retina, cystoid macular edema, outer retinal atrophy, her visual field was reduced to a small infra-nasal isle, reduced scotopic and photopic responses - Others: dizziness, fatigue, cold hands and feet, depression, and sleep disturbances |
|  | **3** | - OPH: decreased visual acuity, and night blindness, bilateral retinal greyish-white pigmentation, left pale optic nerve, intraretinal cysts, extensive outer retinal atrophy, discontinuous ellipsoid zone |
|  | **4** | - OPH: severe visual loss and night blindness, esotropia, pale optic nerve, extensive depigmentation of the retina, occluded retinal vessels, discontinuous ellipsoid zone, extinguished scotopic and photopic responses - HL: Impaired hearing - Other: mental retardation, recurrent papules that manifested on legs and hands |
|  | **5** | - CNS: one encephalitis attack - OPH: pale optic nerves, retinal greyish-white pigmentation, attenuated retinal vessels, reduced scotopic and photopic responses |
| **Kim *et al.***  **2024** | **1** | - OPH: visual impairment sine childhood, temporal pallor of the optic disc in both eyes and mild thinning of the retinal nerve fiber layer - HL: Auditory neuropathy spectrum disorder, difficulty hearing in noisy environments, cochlear nerve deficiency (“atrophy”) |
| **Campbell *et al*.**  **2024** | **1** | - CNS: developmental delay, regression, hypotonia, movement disorder, spasticity |
|  | **2** | - CNS: developmental delay, regression, hypotonia, movement disorder - OPH: vision loss, - HL: hearing loss - Others: nystagmus |
|  | **3** | - CNS: developmental delay, regression, hypotonia, movement disorder, spasticity - OPH: vision loss, optic atrophy, strabismus, retinopathy - Others: nystagmus |
|  | **4** | - CNS: developmental delay, regression, hypotonia, movement disorder OPH: vision loss - Others: nystagmus |
|  | **5** | - CNS: developmental delay, regression, hypotonia, movement disorder, seizure - OPH: vision loss, strabismus - Others: nystagmus |
|  | **6** | - OPH: vision loss, strabismus - HL: hearing loss |
|  | **7** | - CNS: regression, movement disorder - OPH: vision loss, strabismus - HL: hearing loss - Others: nystagmus |
|  | **8** | - CNS: developmental delay, regression, hypotonia, movement disorder - OPH: vision loss - Others: nystagmus |
|  | **9** | - CNS: developmental delay, regression, hypotonia, movement disorder - OPH: vision loss, strabismus |
|  | **10** | - CNS: developmental delay, regression, hypotonia, movement disorder, spasticity, seizure - OPH: vision loss - HL: hearing loss - Others: nystagmus |
|  | **11** | - CNS: developmental delay, hypotonia, movement disorder - OPH: vision loss, strabismus, retinopathy |
|  | **12** | - OPH: vision loss, retinopathy - Others movement disorder |
|  | **13** | - CNS: developmental delay, regression, hypotonia, spasticity, seizure - OPH: vision loss - HL: hearing loss |
|  | **14** | - CNS: developmental delay, hypotonia - OPH: vision loss - Others: nystagmus |
| **Pignatti *et al*.**  **2024** | **1** | - CNS: ataxia, global developmental delay syndrome - PNS: peripheral neuropathy - OPH: optic atrophy - Others: Premature death in the first year of life (infection – respiratory failure) Congenital ambiguous genitalia, androgen excess variation of sex development with normal uterus and ovaries |
|  | **2** |  |
| **Kaler and Couser**  **2024** | **1** | - CNS: cerebral palsy, developmental delay, optic atrophy - PNS: neurogenic bladder, sensorimotor polyneuropathy - OPH: optic atrophy, visual acuity loss, myopia with astigmatism, intermittent exotropia and bilateral ptosis, optic disk pallor - Others: recurrent fractures, scoliosis, bilateral horizontal nystagmus |
| **Cao et *al*.**  **2025** | **1** | - CNS: Ataxia with muscle weakness, unstable gait and frequent falls - OPH: visual acuity of 0.3 in both eyes, pale optic discs, retinal bone spicule-like changes, centrip­etal visual field constriction, thinning of the ganglion cell layer in the macular area on OCT. Optic atrophy and retinitis pigmentosa - HL: Auditory neuropathy |
|  | **2** | - HL: Auditory neuropathy - Others: born prematurely at eight months of pregnancy with a low birth weight, ematemesis, hematochezia, decreased chewing abil­ity and facial pain |
| **Wang et *al*.**  **2025** | **1** | - CNS: unsteady gait from the age of 6 years with symptoms worsening as the disease progressed, Ataxia of limbs, trunk - PNS: decreased pinprick sensation in the distal lower limbs, diminished or absent deep tendon reflexes in the distal lower limbs, low-amplitude sensory responses - OPH: blurred vision from the age of 2 years, visual acuity decreased, retinal pigmentary changes, optic atrophy and retinal vessel attenuation, bilateral diminished retinal nerve fiber layer thickness, bilateral optic atrophy - HL - Others:, exercise intolerance and recurrent lower limb pain during childhood. Cease of menstruation at the age of 16. |
| **Current new reports** | **1** | - PNS: tingling on upper limbs, bilateral low compound muscle action potential amplitudes on common fibular motor nerve - OPH: optic disc atrophy, visual acuity at hand motion retinal dystrophy, vessel narrowing and bone-spicule-like retinal pigmentary changes, binocular kinetic visual field testing showed a small residual island on the right side - HL: Auditory neuropathy spectrum disorder - Others: vestibular bilateral hyporeflexia |
|  | **2** | - OPH: asymptomatic optic atrophy with temporal retinal pallor and thickening - HL: Auditory neuropathy spectrum disorder - Others: vestibular bilateral hyporeflexia, obesity with mellitus diabetes |
|  | **3** | - HL: Auditory neuropathy spectrum disorder, tinnitus - Others: vertigo |
|  | **4** | - PNS: hyposensitivity in the lower limbs with hypopallesthesia and tingling - OPH: asymptomatic optic neuropathy, bilateral temporal pallor, decreased retinal electrogenesis in scotopic and photopic conditions, bilateral axonal deficit - HL: Auditory neuropathy spectrum disorder, tinnitus |
|  | **5** | - CNS: sign of schizophrenia and hallucinations - PNS: feet deficit for dorsal flexion, bilateral positive Babinski signs, and abolition of Patellar tendon and Achilles osteotendinous reflexes. Decreased amplitude of motor and sensitive responses with normal latencies in the lower limbs (axonal anomalies) - OPH: Progressive of visual acuity loss, Bilateral temporal optic atrophy was diagnosed on both eyes associated with central scotoma and dyschromatopsias - HL: Auditory neuropathy spectrum disorder - Others: frequent falls, gait disability |

**Table S1: detailed phenotypes of patients reported up to March 2025**
Clinical signs were categorized as followed: central or peripheral nervous system (CNS, PNS), ophthalmological signs (OPH), hearing loss (HL) and others. Each case number corresponds to that indicated in Table 2.
