# Supplementary material for: Genotype–Phenotype Relationships in Patients Carrying Biallelic FDXR Pathogenic Variants—New Cases and Systematic Review
Source: Hum Mutat. 2026 Oct 2;2026:5928629. doi: 10.1155/humu/5928629 (PMC13633460; doi:10.1155/humu/5928629)
Supplement: Supplementary file 7 [file HUMU-2026-5928629-s007.docx]

|  | **BIALLELIC MISSENSE** (n=49) | | **TRUNCATING + MISSENSE** (n=10) | |
| --- | --- | --- | --- | --- |
|  | Mild (n=18) | Severe (n=31) | Mild (n=4) | Severe (n=6) |
| **NADPH1** | 1 (4%) | 1 (2%) | 0 (0%) | 2 (29%) |
| **FAD** | 16 (59%) | 10 (23%) | 3 (75%) | 3 (43%) |
| **NADPH2** | 1 (4%) | 20 (47%) | 1 (25%) | 2 (29%) |

**Table S2 : Mild and severe phenotypes among patients carrying either biallelic missense variants in one same binding-domain, and those with truncating associated with missense variants**
Patients carrying biallelic missense variants analyzed in this table were those classified in the NADPH1, FAD and NADPH2 groups, for a consistent comparison with the missense variant of the other group. Patients carrying truncating variants were those described by Paul (patient 5), Peng (patient 12 and 14), Jurkute (one of the two siblings patients 2 and 3), Campbell (patients 5, 12 and 14), Wang (patient 1), Cao (patient 1), Slone (patient 2) and Wei (patients 1 and 3). In both groups, variants in FAD domain seemed to be more related to mild phenotypes, contrary to those in NADPH2.
